# Evaluation of Mucosal Healing in Ulcerative Colitis by Fecal Calprotectin vs. Fecal Immunochemical Test: A Systematic Review and Meta-analysis

**DOI:** 10.5152/tjg.2023.22812

**Published:** 2023-09-01

**Authors:** Tingpeng Hu, Zhimei Zhang, Fusheng Song, Wenguang Zhang, Jun Yang

**Affiliations:** Department of Gastroenterology, Banan Hospital of Chongqing Medical University, Chongqing, China

**Keywords:** Ulcerative colitis, fecal immunochemical test, fecal calprotectin, diagnostic accuracy, mucosal healing

## Abstract

Mucosal healing has been considered a treatment goal for patients with inflammatory bowel disease. To compare the accuracy of fecal immunochemical test and fecal calprotectin in the judgment of mucosal healing in ulcerative colitis, a meta-analysis was performed. We searched the PubMed, Cochrane Library, Web of Science, and Embase for the studies on fecal immunochemical test and fecal calprotectin predicting mucosal healing in ulcerative colitis. The comprehensive sensitivity, specificity, diagnostic odds ratio, positive likelihood ratio, and negative likelihood ratio were calculated to evaluate the accuracy. By analyzing 22 publications, we found that the combined sensitivity and specificity of fecal immunochemical test were 0.87 (95% CI, 0.80-0.92) and 0.73 (95% CI, 0.62-0.81), respectively. The combined sensitivity and specificity of fecal calprotectin were 0.76 (95% CI, 0.70-0.80) and 0.80 (95% CI, 0.76-0.84), respectively. The area under the curve values of the fecal immunochemical test and fecal calprotectin summary receiver operating characteristic (SROC) curves were 0.88 and 0.85, respectively. Consequently, fecal immunochemical test had higher sensitivity in predicting mucosal healing in ulcerative colitis patients, while fecal calprotectin had higher specificity. Compared with fecal calprotectin, fecal immunochemical test was more accurate in judging mucosal healing in ulcerative colitis.

**Main Points**

- Mucosal healing (MH) can achieve a sustained clinical remission in ulcerative colitis.
- This meta-analysis compared fecal immunochemical tests with fecal calprotectin for evaluating MH.
- The area under the curve value showed that fecal immunochemical tests had a higher diagnostic performance.
- It is important to identify the optimal cutoff values of the biomarkers in the future.

## Introduction

Ulcerative colitis (UC), 1 subtype of inflammatory bowel disease, is an idiopathic chronic inflammatory disease, and its incidence is increasing worldwide.^1,2^ The clinical manifestations of UC are mainly stomachache, diarrhea, hematochezia, and weight loss. Due to the relapsing-remitting course of UC, it is particularly important for patients to achieve its therapeutic targets. In recent years, the therapeutic targets of UC have been upgraded from clinical remission to mucosal healing (MH).^3^ Mucosal healing can not only achieve a sustained clinical remission but also reduce the risk of recurrence, the hospitalization rate and the surgical resection rate.^4^ At present, colonoscopy is the gold standard for evaluating MH, but it is not practical because of its invasiveness and risk of complications, as well as its expensive and time-consuming nature.^5^ Finding reliable noninvasive biomarkers to predict MH has become a research hotspot in recent years.

C-reactive protein, white blood cell ratio, and other serum markers can indicate the systemic inflammatory response but lack specificity for intestinal inflammation. Stool markers have good sensitivity and specificity for detecting intestinal inflammation. In addition, fecal markers can well reflect the inflammatory status of the mucosa and be used to evaluate the progress of the patients.^6^ Fecal calprotectin (FC) and fecal immunochemical tests (FITs) are common markers of stool. Fecal calprotectin is a white blood cell protein of the S100 family that mainly exists in human neutrophils. When FC is released into the extracellular space, neutrophils migrate to the site of inflammation and produce phagocytic activity. Fecal calprotectin is an acute phase reactive protein. When intestinal inflammation occurs, FC is released in large quantities into the intestine, and finally, its concentration in feces increases. Therefore, FC can not only reflect the infiltration of neutrophils in the intestinal mucosa but also be used to evaluate the severity of intestinal inflammation. Fecal calprotectin is also very sensitive in evaluating therapeutic efficacy and predicting disease recurrence.^7,8^ Fecal immunochemical test is a sensitive, inexpensive, and rapid method to measure the concentration of hemoglobin in feces by using a human hemoglobin antibody. Fecal immunochemical test is recognized as the important biomarker for colorectal cancer screening in most countries.^9,10^ For the past few years, many studies have proven that FIT is also extremely sensitive to the detection of inflammatory bowel diseases (IBD) such as mucosal ulcers and hidden blood loss.

Although both high FIT and FC are sensitive for the discovery of mucosal inflammation in IBD, the diagnostic efficacy of low FIT and/or FC in MH has not been conclusive. If we can find a stool marker that shows a high sensitivity and specificity for detecting MH, it may be a promising alternative to invasive endoscopic monitoring of patients with IBD who are intolerant to endoscopy or who are unsuitable for repeated endoscopy. This study aims to compare the accuracy of FIT and FC in the assessment of MH in UC patients by systematically reviewing the relevant literature.

## Materials and Methods

This meta-analysis was carried out and reported on the basis of the statement on Preferred Reporting Items for Systematic Reviews and Meta-Analyses.^11^

### Literature Search

We searched databases including PubMed, Cochrane Library, Web of Science, Embase, and Medline for studies on FC and/or FIT detection of UC MH. Gray literature was excluded in the retrieval process. The search terms included FIT, fecal calprotectin or FC, inflammatory bowel disease or IBD or ulcerative colitis or UC or colitis or enteritis or intestinal inflammation, sensitivity, and specificity. The retrieval time was from the establishment time of each database to July 2021. To avoid omitting qualified studies, the references of relevant literature were manually searched.

### Study Selection

The literature was screened independently by 2 researchers. First, 2 researchers screened the articles according to their titles and abstracts and then further screened the articles that might meet the requirements by reading the full text. Any discrepancies in the study selection process were discussed and resolved with the third researcher. The inclusion criteria were as follows: (i) the subjects were patients diagnosed with UC; (ii) all patients underwent FC and/or FIT tests to predict MH in UC patients; (iii) the endoscope scoring system was used as the reference standard for MH evaluation; and (iv) sufficient raw data could be extracted directly or indirectly, including true-positive (TP), true-negative (TN), false-positive (FP), and false-negative (FN) values. We excluded articles with incomplete data, duplicate articles, animal studies, reviews, and other irrelevant studies.

### Data Extraction

Two researchers independently extracted data from each included study and reached an agreement after discussion with the third researcher when disagreements occurred. The extracted data included (i) the first author, publication year, and country; (ii) number of patients, age of the patients, reference criteria of MH, cutoff values of FC, and FIT; and (iii) TP, FP, FN, and TN values of each included study.

### Methodological Quality Assessment

The methodological quality of each included study was assessed using the Diagnostic Accuracy Quality Evaluation Study Table (QUADAS-2). The QUADAS-2 tool includes 4 key areas: patient selection, index testing, reference standards, and process and time. Each area was evaluated according to the risk of bias.^12^ The QUADAS list consists of 14 items, and each item is judged as “yes” (low degree of deviation or good applicability), “no” (high degree of deviation or poor applicability), and “unclear” (lack of relevant information or uncertainty of deviation). Finally, Review Manager 5.3 statistical software was used to draw a deviation risk map.

### Statistical Analysis

Meta-disc 1.4 and Stata 15.0 statistical software were used for data analysis. The combined sensitivity, specificity, area under the curve (AUC), diagnostic odds ratio (DOR), positive likelihood ratio (PLR), negative likelihood ratio (NLR), and CI were calculated according to the 2 × 2 contingency table of each included study. The final comparison indicator was AUC. If the AUC is close to 1, the test is good; if the AUC is close to 0.5, the test is poor. The Spearman correlation coefficient was used to test the threshold effect. If *P* > .05, there is no threshold effect among the studies. Heterogeneity between different studies was assessed by the chi-square test and *I*^2^ statistics. When *I*^2^ >50%, the heterogeneity was significant, and the random-effects model was used to estimate the results. When *I*^2^ <50%, the heterogeneity was not significant, and a fixed-effects model was used to estimate the results. Meta-regression and sensitivity analysis were used to assess possible explanations for significant heterogeneity. Publication bias was assessed by the Deek’s test. *P* < .05 indicates that there is publication bias in the included studies.

## Results

### Study Selection

**Figure 1.**
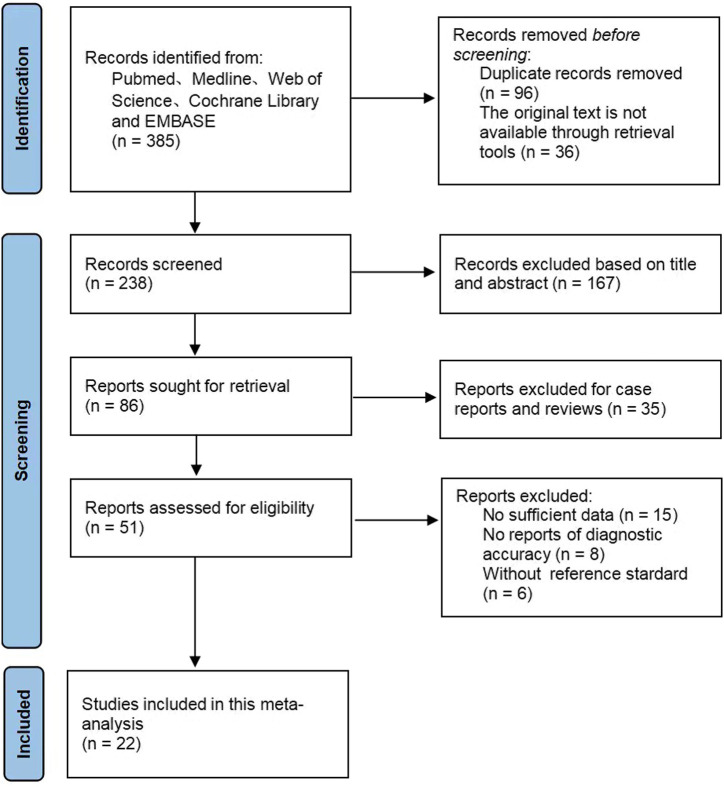
Flowchart of the literature search.

After the first retrieval, a total of 385 publications were obtained, and after the removal of 96 duplicates, 361 citations remained. Then, based on the article titles and abstracts, 167 irrelevant documents were excluded. After reading the full text of the remaining articles, 22 articles^13-34^ (37 studies) were finally included, including 3541 patients. The literature retrieval flowchart is shown in Figure 1.

### Characteristics of the Included Studies

Of the 22 included publications (37 studies), 4 publications (4 studies) detected only FIT,^16,18-20^ 12 publications (23 studies) detected only FC,^23-34^ and 6 publications (10 studies) simultaneously tested FIT and FC.^13-15,17,21,22^ The reference criteria for assessing MH in the included studies were not entirely consistent, but they were all based on the endoscopic scoring system. Among them, 31 studies took the Mayo endoscopic score (MES) as the reference standard, 4 studies used the ulcerative colitis endoscopy severity index (UCEIS), 1 study used the modified PICaSSO endoscopy score (modPICaSSO), and 1 study used the Rachmilewitz index (RI). In 14 FIT studies, the cutoff value of FIT ranged from 10 to 280 ng/mL. The patients participating in the study were between 3 and 81 years old. The characteristics of the included studies are shown in Tables 1 and 2.

### Quality Assessment

**Figure 2.**
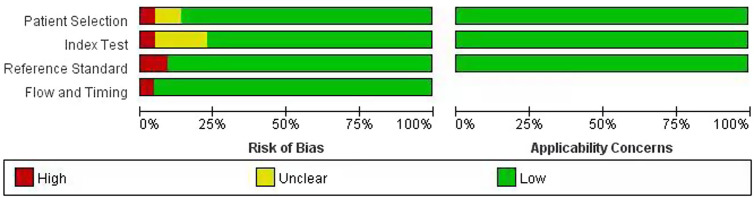
Quality analysis of the included studies based on the QUADAS-2 criteria.

All of the included studies passed quality evaluation by adopting the abovementioned QUADAS-2 tool, and the results indicated that the whole quality of the included studies was good. All study subjects were considered to be a representative spectrum of patients. The largest difference among the studies was the difference in MH reference standards, including the MES, UCEIS, modPICaSSO, and RI. In addition, these studies had high applicability to the 3 areas of patient selection, indicator tests, and gold standards. No studies were excluded after completing the quality evaluation. Figure 2 shows the details of the quality assessment.

### Accuracy of Fecal Immunochemical Test and Fecal Calprotectin

**Figure 3.**
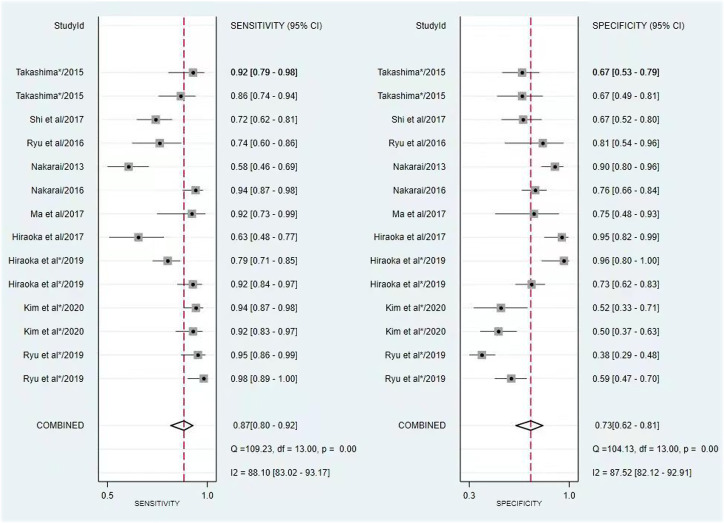
Forest plot of the sensitivity and specificity of FIT to predict MH. FIT, fecal immunochemical test; MH, mucosal healing.

**Figure 4.**
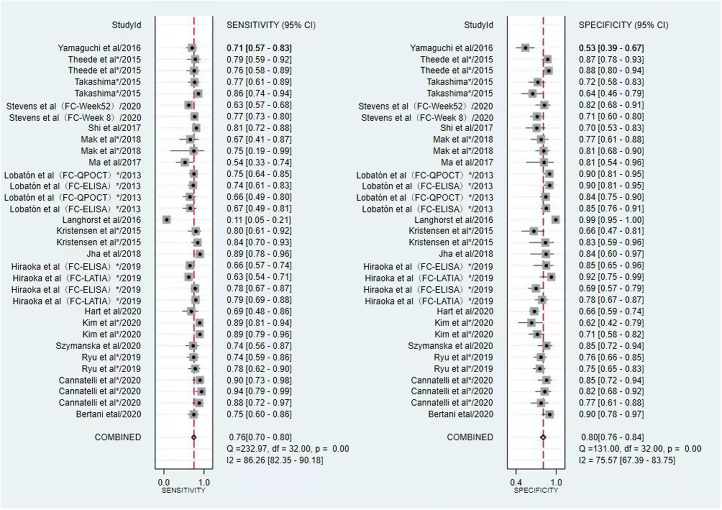
Forest plot of sensitivity and specificity of FC to predict MH. FC, fecal calprotectin; MH, mucosal healing.

**Figure 5.**
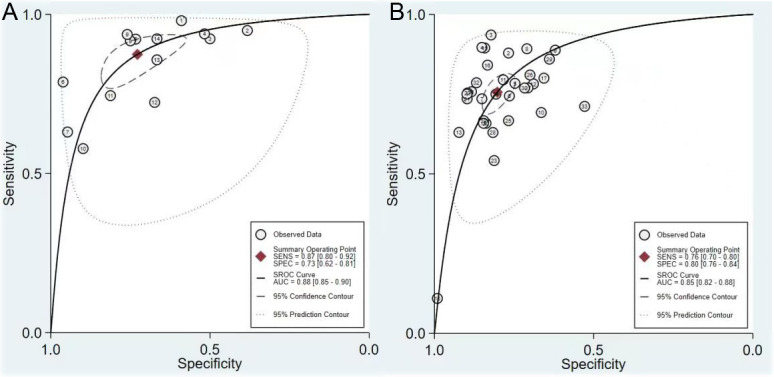
SROC to predict mucosal healing of ulcerative colitis by FIT (A) and FC (B). FC, fecal calprotectin; FIT, fecal immunochemical test.

The pooled sensitivity, specificity, PLR, NLR, and DOR of MH in patients with UC assessed by FIT were 0.87 (95% CI, 0.80-0.92), 0.73 (95% CI, 0.62-0.81), 3.21 [95% CI, 2.36-4.38], 0.17 [95% CI, 0.12-0.25], and 18.60 [95% CI, 12.49-27.69], respectively (Figure 3). The comprehensive sensitivity, specificity, PLR, NLR and DOR of MH in patients with UC assessed by FC were 0.76 [95% CI, 0.70-0.80], 0.80 [95% CI, 0.76-0.84], 3.83 [95% CI, 3.23-4.55], 0.30 [95% CI, 0.26-0.36], 12.63 [95% CI, 10.00-15.96], respectively (Figure 4). In predicting MH in UC, FIT had a higher sensitivity, while FC had a higher specificity. The AUC value of the SROC curve showed that FIT had a higher diagnostic performance (Figure 5).

### Heterogeneity Test

The statistical results displayed that the sensitivity (*I* ^2^ = 88.10, 86.26) and specificity (*I* ^2^ = 87.52, 75.57) of FIT and FC had high heterogeneity. The Spearman’s correlation coefficient and *P* value of FIT and FC were 0.741 (*P* = .002, *P* < .05) and 0.324 (*P* = .066, *P* > .05), respectively. These results indicated that there was a threshold effect in the FIT group but not in the FC group. The nonthreshold effect was evaluated by meta-regression analysis in the FIT group and FC group. And what this shows is that the judgment method of MH, the cutoff value, and the number of patients enrolled had no prominent impact on heterogeneity in the FIT prediction of MH in UC (Table 3). In the study of FC prediction of MH in UC, the number of patients included was one of the reasons for the heterogeneity, while the region, definition of MH, and cutoff value had no significant effect on heterogeneity (Table 4).

### Publication Bias

**Figure 6.**
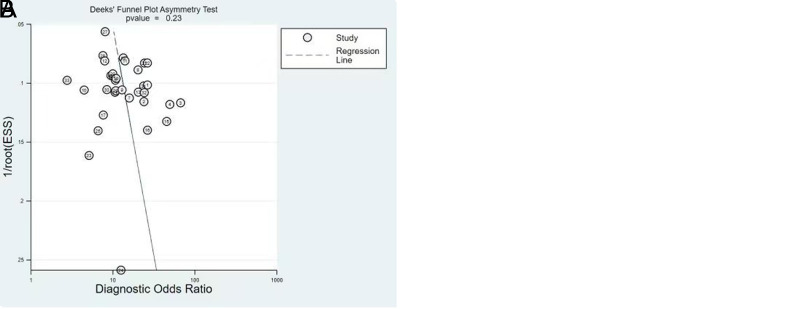
Deek’s funnel plot of FIT (A) and FC (B). FC, fecal calprotectin; FIT, fecal immunochemical test.

The Deek’s test showed that the *P* values of FIT and FC were 1.00 and .23, respectively. This indicated that the 14 FIT studies and 33 FC studies had no publication bias (Figure 6).

## Discussion

Ulcerative colitis is a common disease encountered in the clinic. In recent years, with continuous changes in living habits and diet structure, the disease has shown a trend of increased incidence worldwide, and the main manifestation is a chronic intestinal nonspecific inflammatory reaction, which has the characteristics of a long course of disease and recurrent symptoms.^35^ Since the disease course of UC is characterized by multistage clinical remission and acute exacerbation, it is necessary to continue to pay attention to the condition and treatment effect of UC patients. Determining disease activity during the asymptomatic stage is of consequence for predicting the recurrence of the disease. At present, MH has been identified as the main treatment target for UC.^36^ Studies have shown that MH can reduce the recurrence rate, hospitalization rate, and operation rate of the patients. The evaluation of MH has become an important part of the treatment and follow-up of UC patients.^37^ Although endoscopy is the gold standard for evaluating MH, frequent endoscopy is not only costly but also increases the risk of complications for patients. It is the common goal of researchers to find biomarkers that can accurately, quickly, and noninvasively predict MH.

Commonly used stool markers include FC and FIT. Fecal calprotectin estimates the severity of intestinal inflammation according to the number of inflammatory cells, while FIT measures the release of blood from the damaged intestinal mucosa. Several studies have shown that both FC and FIT can recognize the disease activity of UC patients similar to endoscopy, and both are promising noninvasive markers of mucosal inflammation.^38-40^ Because FC is expensive, it has not been popularized in most countries. In contrast, FIT is cheap. From an economic point of view, FIT is better for repeated testing in UC patients than FC. Compared with FIT, FC also has the disadvantages of a long time-consuming and cumbersome detection process. Fecal calprotectin is analyzed by an enzyme-linked immunosorbent assay, which required 5-10 g of fecal material. The FC detection process also requires professional technicians to operate and often takes several hours to complete.^39^ However, the fecal samples of FIT can be analyzed automatically after being loaded into the kit, which does not require any other professional operations and takes only a short time. Detection can often be completed in a few minutes.^41^ The disadvantage of FIT is that it measures intestinal hemoglobin rather than specific indicators of intestinal inflammation.

The goal of this study was to compare the diagnostic value of FC and FIT in predicting MH in patients with UC by meta-analysis. This study included 22 studies (37 studies) with 3541 patients in total. The quality evaluation results suggested that the overall quality was relatively high. The results of this study showed that the combined sensitivity and specificity of the 14 FIT studies were 0.87 (95% CI, 0.80-0.92) and 0.73 (95% CI, 0.62-0.81), respectively, and the AUC was 0.88. The combined sensitivity and specificity for FC were 0.76 [95% CI, 0.70-0.80] and 0.80 [95% CI, 0.76-0.84], respectively, and the AUC was 0.85. These results indicated that FIT had higher sensitivity in determining MH of UC, while FC had a higher specificity. The AUC value showed that FIT had a higher diagnostic performance.

In this meta-analysis, high heterogeneity was found in both the FC and FIT groups. Therefore, we conducted meta-regression analysis on 33 FC studies and 14 FIT studies. The covariables in the meta-regression analysis of the FC group included the following: (i) district (Asian or European or American); (ii) definition of healing (MES = 0 or MES ≤ 1 or others); (iii) number of patients included in the study (n < 100 or 200 > n ≥ 100 or n ≥ 200); and (iv) cutoff (µg/g) (cutoff < 100 or 200 > cutoff ≥ 100 or cutoff ≥ 200). The results indicated that the number of patients enrolled in the study was one of the reasons for the heterogeneity, but none of the other covariables mentioned above was the reason for the heterogeneity. Covariables of meta-regression analysis in the FIT group included (i) the definition of superiority healing (MES = 0 or MES ≤ 1 or others); (ii) cutoff (µg/g) (cutoff < 100 or cutoff = 100 or others); (iii) number of patients included in the study (n < 100 or N ≥ 100). The results displayed that the above covariates are not the cause of heterogeneity. However, due to the limited sample size, we cannot further analyze any other factors that may have caused the heterogeneity, such as the measurement methods of FC and FIT, the age of the patients and the treatment plan of UC.

There are some limitations to this meta-analysis. First, we failed to contact the authors of each study to obtain more information about the clinical features of the patients included in each study, such as the course and severity of the UC involving intestinal segments, which may be one of the reasons for the heterogeneity. Second, the cutoff values of FC and FIT in the various studies were not consistent, and there were obvious fluctuations. Our meta-analysis cannot provide a clear cutoff value of FC and FIT for clinical reference. Therefore, more high-quality, large-scale prospective studies are needed to verify the diagnostic value of FC and FIT in the future to predict MH in patients with UC and to identify the optimal cutoff values of FC and FIT.

## Conclusion

Our research showed that FIT had higher sensitivity in predicting MH in UC patients, while FC had higher specificity. The AUC value of the SROC curve showed that both FIT and FC had high diagnostic performance and that the diagnostic performance of FIT was higher. Therefore, we believe that both FIT and FC are reliable noninvasive markers for predicting MH in UC patients and can replace invasive endoscopy in some patients.

## Figures and Tables

**Table 1.** Characteristics of the Included Studies (FIT)

Study	Year	Country	Age	Mucosal Healing Definition	Sample size (n)	Sensitivity (%)	Specificity (%)	Cutoff (ng/mL)
Ryu et al^13*^	2019	Korea	47.2 ± 13.8	MES = 0	174	98.00	37.40	100
Ryu et al^13*^	2019	Korea	47.2 ± 13.8	UCEIS ≤ 1	174	94.90	38.30	100
Kim et al^14*^	2020	Korea	50 (18-81)	MES 0	127	92.30	50	10
Kim et al^14*^	2020	Korea	50 (18-81)	MES ≤ 1	127	93.90	51.70	60
Hiraoka et al (FIT-LATIA)^15*^	2019	Japan	44 (14-78)	MES 0	152	92.00	73.00	50
Hiraoka et al (FIT-LATIA)^15*^	2019	Japan	44 (14-78)	MES ≤ 1	152	79.00	96.00	78
Hiraoka et al^16^	2017	Japan	33 (9-77)	MES = 0	84	63.00	95.00	100
Ma et al^17^	2017	Canada	45.5 (30.9-57.5)	UCEIS = 3	40	92.00	75.00	100
Nakarai^18^	2016	Japan	32 (22-43)	MES 0/1	194	94.00	76.00	100
Nakarai^19^	2013	Japan	30 (4-80)	MES 0/1	152	58	90	60
Ryu et al^20^	2016	Korea	47.9 (22-75)	MES 0/1	63	73	81	100
Shi et al^21^	2017	China	50 (38-61)	MES 0/1	140	72	68	50
Takashima^22^*	2015	Japan	35.5 (14-77)	MES 0/1	92	86	66	280
Takashima^22^*	2015	Japan	35.5 (14-77)	MES = 0	92	93	67	75

^*^Different studies from the same literature.

FIT, fecal immunochemical test; MES, Mayo endoscopic score; UCEIS, ulcerative colitis endoscopic index of severity.

**Table 2.** Characteristics of the Included Studies (FC)

Study	Year	Country	Age	Mucosal Healing Definition	Sample size (n)	Sensitivity (%)	Specificity (%)	Cutoff
Bertani etal^23^	2020	Italy	46.5 ± 14.6	MES ≤ 1	97	0.75	0.889	157.5 mg/kg
Cannatelli et al^24*^	2020	UK	44.2 ± 14.5	modPICaSSO ≤ 3	76	87.90	76.70	161 mcg/g
Cannatelli et al^24*^	2020	UK	44.2 ± 14.5	UCEIS ≤ 1	76	93.50	82.20	148 mcg/g
Cannatelli et al^24*^	2020	UK	44.2 ± 14.5	MES = 0	76	89.70	85.10	112 mcg/g
Ryu et al^13*^	2019	Korea	47.2 ± 13.8	MES = 0	128	78.40	74.80	170 μg/g
Ryu et al^13*^	2019	Korea	47.2 ± 13.8	UCEIS ≤ 1	128	74.60	76.50	170 μg/g
Szymańska et al^25^	2020	Poland	3-18	MES = 0	81	74.00	85.00	105 µg/g
Kim et al^14*^	2020	Korea	50 (18-81)	MES 0	127	89.20	71	70 μg/g
Kim et al^14*^	2020	Korea	50 (18-81)	MES ≤ 1	127	88.80	62.10	200 μg/g
Hart et al^26^	2020	Canada	48 ± 13.9	MES ≤ 1	185	69.00	65.00	170 mcg/g
Hiraoka et al (FC-LATIA)^15*^	2019	Japan	44 (14-78)	MES 0	152	79.00	78.00	224 μg/g
Hiraoka et al (FC-ELISA)^15*^	2019	Japan	44 (14-78)	MES 0	152	78.00	69.00	184 μg/g
Hiraoka et al (FC-LATIA)^15*^	2019	Japan	44 (14-78)	MES ≤ 1	152	63.00	92.00	251 μg/g
Hiraoka et al (FC-ELISA)^15*^	2019	Japan	44 (14-78)	MES ≤ 1	152	66.00	85.00	204 μg/g
Jha et al^27^	2018	India	35 (14-60)	MES = 0	76	90.00	85.00	158 μg/g
Kristensen et al^28*^	2015	Norway	35.5 (18-72)	MES = 0	62	84.10	83.30	62μg/g
Kristensen et al^28*^	2015	Norway	35.5 (18-72)	MES ≤ 1	62	80.00	66.60	110 μg/g
Langhorst et al^29^	2016	Germany	48.1 ± 13.4	RI ≤ 1	174	10.90	99.00	13.9 µg/g
Lobatón et al (FC-ELISA)^30*^	2013	Spain	47 ± 17	MES = 0	146	66.70	84.50	160 µg/g
Lobatón et al (FC-QPOCT)^30*^	2013	Spain	47 ± 17	MES = 0	146	64.90	83.90	161 μg/g
Lobatón et al (FC-ELISA)^30*^	2013	Spain	47 ± 17	MES ≤ 1	146	73.50	89.70	250 μg/g
Lobatón et al (FC-QPOCT)^30*^	2013	Spain	47 ± 17	MES ≤ 1	146	75.40	89.10	280 μg/g
Ma et al^17^	2017	Canada	45.5 (30.9-57.5)	UCEIS = 3	40	54.00	81.00	250 μg/g
Mak et al^31*^	2018	USA	29.3 ± 17.2	MES = 0	61	75.00	80.00	200 μg/g
Mak et al^31*^	2018	USA	29.3 ± 17.2	MES ≤ 1	61	67.00	77.00	250 μg/g
Shi et al^21^	2017	China	50 (38-61)	MES 0/1	140	81	71	155 μg/g
Stevens et al (FC-Week 8)^32^	2020	Netherlands	—	MES ≤ 1	639	77	69	251 μg/g
Stevens et al (FC-Week52)^32^	2020	Netherlands	—	MES ≤ 1	373	63	81	99 μg/g
Takashima^22*^	2015	Japan	35.5 (14-77)	MES 0/1	92	86	63	369 μg/g
Takashima^22*^	2015	Japan	35.5 (14-77)	MES = 0	92	77	72	200 μg/g
Theede et al^33*^	2015	Denmark	41 (19-76)	MES = 0	120	75	88	192 mg/kg
Theede et al^33*^	2015	Denmark	41 (19-76)	UCEIS = 0	120	79	87	192 mg/kg
Yamaguchi et al^34^	2016	Japan	45 (36-54)	MES = 0	105	71	53	194 μg/g

^*^Different studies from the same literature.

FC, fecal calprotectin; ELISA, enzyme-linked immunosorbent assay; MES, Mayo endoscopic score; modPICaSSO, modified PICaSSO endoscopy score; RI, Rachmilewitz index; UCEIS, ulcerative colitis endoscopic index of severity.

**Table 3.** Results of the Meta-regression Analysis of 14 FIT Studies

Parameter	Coefficient	SE	*P*
The definition of mucosal healing (MES = 0 or MES ≤ 1 or Others)	–0.308	0.33	.373
Cutoff (μg/g) (cutoff < 100 or cutoff = 100 or others)	–0.365	0.332	.297
Number of patients included in the study (n < 100 or n ≥ 100)	0.236	0.417	.584

FIT, fecal immunochemical test; MES, Mayo endoscopic score.

**Table 4.** Results of the Meta-regression Analysis of 33 FC Studies

Parameter	Coefficient	SE	*P*
District (Asian or European or American)	0.143	0.159	.375
The definition of mucosal healing (MES = 0 or MES ≤ 1 or others)	–0.129	0.138	.357
Number of patients included in the study (n < 100 or 200 > n ≥ 100 or n ≥ 200)	–0.395	0.159	.019
Cutoff (μg/g) (cutoff < 100 or 200 > cutoff ≥ 100 or cutoff ≥ 200)	–0.138	0.138	.325

FC, fecal calprotectin; MES, Mayo endoscopic score.
